# Extract of *Deschampsia antarctica* (EDA) Prevents Dermal Cell Damage Induced by UV Radiation and 2,3,7,8-Tetrachlorodibenzo-p-dioxin

**DOI:** 10.3390/ijms20061356

**Published:** 2019-03-18

**Authors:** Alicia Zamarrón, Esther Morel, Silvia Rocío Lucena, Manuel Mataix, Azahara Pérez-Davó, Concepción Parrado, Salvador González

**Affiliations:** 1 Department of Biology, Faculty of Sciences, Autónoma University of Madrid, IRYCIS, 28049 Madrid, Spain; aliszm@gmail.com (A.Z.); silvialucenablas@gmail.com (S.R.L.); manuel.mataix@estudiante.uam.es (M.M.); 2 Animal Health Research Center (CISA-INIA), Valdeolmos, 28130 Madrid, Spain; embarcena@gmail.com; 3 Department of Basic Health Sciences, Faculty of Health Sciences, Rey Juan Carlos University, Alcorcón, 28922 Madrid, Spain; 4 Cantabria Labs, 28043 Madrid, Spain; azahara.perez@cantabrialabs.es; 5 Department of Histology and Pathology, University of Málaga, 29071 Málaga, Spain; cparrado@uma.es; 6 Department of Medicine and Medical Specialties, Alcalá de Henares University, 28805 Madrid, Spain

**Keywords:** photoaging, ultraviolet light, dioxins, natural extract, in vitro studies, dermal fibroblast, keratinocytes

## Abstract

Exposure to natural and artificial light and environmental pollutants are the main factors that challenge skin homeostasis, promoting aging or even different forms of skin cancer through a variety of mechanisms that include accumulation of reactive oxygen species (ROS), engagement of DNA damage responses, and extracellular matrix (ECM) remodeling upon release of metalloproteases (MMPs). Ultraviolet A radiation is the predominant component of sunlight causative of photoaging, while ultraviolet B light is considered a potentiator of photoaging. In addition, different chemicals contribute to skin aging upon penetration through skin barrier disruption or hair follicles, aryl hydrocarbon receptors (AhR) being a major effector mechanism through which toxicity is exerted. *Deschampsia antarctica* is a polyextremophile Gramineae capable of thriving under extreme environmental conditions. Its aqueous extract (EDA) exhibits anti- photoaging in human skin cells, such as inhibition of MMPs, directly associated with extrinsic aging. EDA prevents cellular damage, attenuating stress responses such as autophagy and reducing cellular death induced by UV. We demonstrate that EDA also protects from dioxin-induced nuclear translocation of AhR and increases the production of loricrin, a marker of homeostasis in differentiated keratinocytes. Thus, our observations suggest a potential use exploiting EDA’s protective properties in skin health supplements.

## 1. Introduction

The skin is an organ specialized as a first major barrier against ionizing radiations and chemical damage. Its complete exposure to environmental DNA damaging agents renders it susceptible of developing different pathologies, including cancer. Skin tumours are the most common form of cancer, and its deadliest type—melanoma—exhibits an increasing incidence and accounts for ~1% of all cancer-related deaths worldwide (World Health Organization Annual Report, 2017).

Ultraviolet ionizing radiation (UVR) constitutes ~10% of total sunlight output, and is the most prominent causative factor for skin cancers (reviewed in open online resources: In. *Ultraviolet Waves*.; 2010). Its long- and medium-wave components A and B (UVA, wavelength 320–400 nm and UVB, wavelength 290–320 nm)—those most penetrant through the ozone layer—can induce DNA damage directly, by promoting the formation of specific DNA products (e.g., pyrimidine-pyrimidine dimers, most often formed by the crosslinking of thymine pairs). Moreover, telomeres are particularly sensitive to UVR damage due to their relative enrichment in target TT and G bases, amplifying the genome instability resulting from genome-wide point alterations [1]. In addition, UVR can damage DNA and other cell structures through the accumulation of reactive oxygen species (ROS), which in turn favour further damage upon activation of inflammatory signalling and disruption of mitochondrial function. Surviving unrepaired damaged cells can initiate tumours or contribute to skin aging (reviewed in [2,3]).

Of note, a number of chemicals can non-specifically enhance the toxicity of skin cells by rising basal metabolic/oxidative stress. A prominent example is embodied by dioxins, highly toxic, persistent organic pollutants of which 2,3,7,8-Tetrachlorodibenzo-p-dioxin (TCDD) is a prototypical species. Dioxins exert their toxicity by acting as potent ligands of the bHLH transcriptional regulator aryl hydrocarbon receptor (AhR), a pivotal xenobiotic sensor in eukaryotic cells that, upon activation, can alter global gene expression patterns, and trigger metabolic programmes that amplify the accumulation of toxic compounds [4].

Given current habits favouring outdoor activities and the pervasiveness of pollutants with phototoxic potential, identifying natural compounds with potential protective activity is warranted. The Antarctic hair grass *Deschampsia antarctica* is a tracheophyte capable of thriving under extreme weather conditions, including high oxygen tension and solar radiation [5]. One of only two flowering plants in Antarctica, it partly owes its resilience to secondary metabolism routes, which provide photoquenching compounds as well as phenolic substances with strong antioxidant potential, including flavonoids such as apigenin and luteolin [6]. Previous studies on soluble extracts of *Deschampsia antarctica* (hereon EDA) support that these activities can be transferred as antioxidant and antiaging properties on human cells [7]. Therefore, these preparations have the potential to be used as protective supplements against environmental aggressions, but the characterization of their specific activity in the face of specific agents is still lacking.

Matrix metalloproteinases (MMPs) constitute a heterogeneous family of enzymes capable of hydrolyzing collagen and degrading different components of the ECM, and are involved in several physiological and pathological processes. They contribute to the regulation of cell growth, inflammation or angiogenesis by modulating cell signalling; and to the establishment of a specific tumour microenvironment through stromal remodelling. Their activity is tightly regulated by endogenous inhibitors the tissue inhibitor of metalloproteinases (TIMPs). The activity of MMPs has been specifically associated with photoageing [8]. A prominent member is the collagenase MMP1, an ubiquitous, potent MMP capable of degrading collagens I, II and III that is upregulated by different sources of cell stress [9].

Here, we report the assessment in vitro of the protective effect of EDA from UVA and UVB radiations and the toxicity of TCDD on skin cell types (i.e., skin fibroblasts and keratinocytes). Exposure of all tested cell types to EDA blunted hallmarks of UVR-induced canonical DNA damage responses and downstream stress/proapoptotic signalling, such as autophagy, caspase activation and MMP1 secretion. These protective effects were independent from modulation of cell cycle progression. Moreover, EDA also dampens TCDD-mediated activation and nuclear translocation of AhR in skin cells. Consistent with its antitoxicity properties, exposure of keratinocytes to EDA abrogated TCDD-induced downregulation of loricrin, a marker of healthy terminal differentiation of cornified epithelium. Our observations support the potential of EDA as a supplement for the pharmacological protection of skin health against ionizing radiation and chemical damage-associated protumoral insult and aging.

## 2. Results

### 2.1. EDA does not have Apparent Effects on Healthy Fibroblasts and Keratinocytes, but Reverts Alterations Induced by UV Radiation

**Figure 1:**
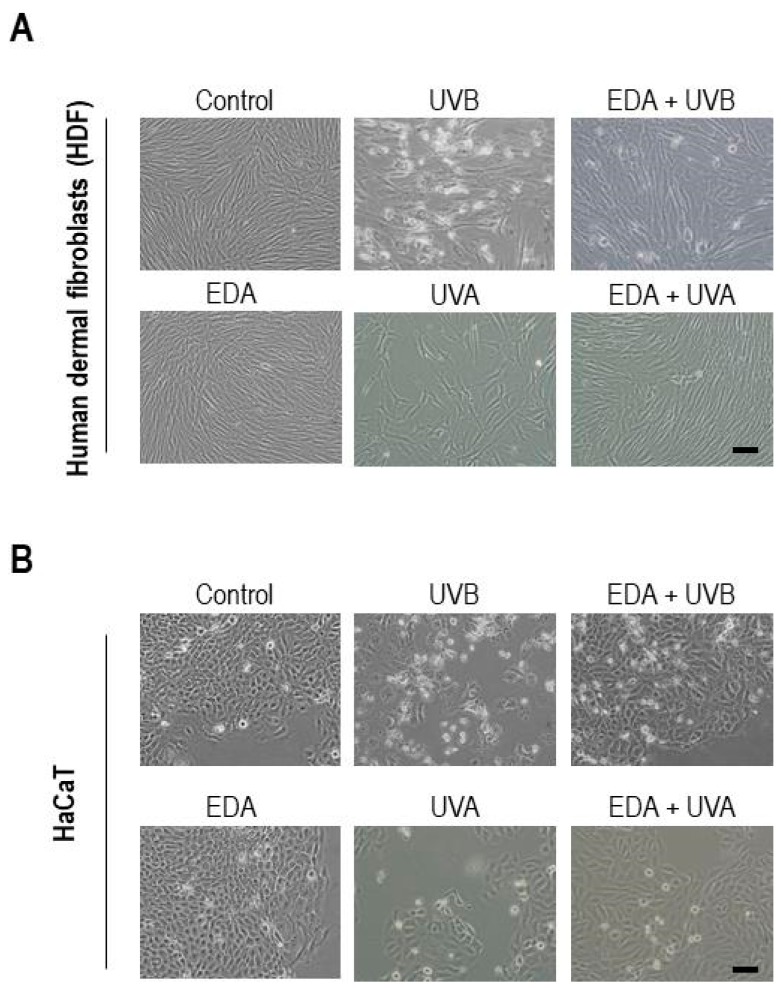
Cell morphology of HDF and HaCaT keratinocytes after treatment with EDA and/or UV radiation. HDF (**A**) and HaCaT cells (**B**) were incubated with 0.5 mg/mL EDA for 24 h and then irradiated with UVB (700 mJ/cm^2^ for HDF and 300 mJ/cm^2^ for HaCaT cells) or UVA (3000 mJ/cm^2^). Cultures were observed at 48 h after treatment and images were captured. Scale bar: 50 μm.

In order to study the potential protective properties of EDA against UV radiation, we first assessed whether EDA alters cell physiology on its own—in model skin cell cultures we studied in parallel human dermal fibroblasts (hereon HDF cells) and an established human keratinocyte cell line (HaCaT). Exposure of HDF or HaCaT cells to EDA alone for 24 h did not impact visibly on cell culture morphology and confluency (Figure 1A,B, leftmost panels), and had no detrimental effect on cell proliferation (Figure S1). In fact we did observe a modest yet significant increase in cell proliferation when HDF cells were supplemented with EDA for either 24 or 48 h (Figure S1). These observations suggest that EDA does not have a major impact on the physiology of healthy cells in culture. Next, we performed dose-response curves to identify the lowest radiation dose that would exert a detectable, reproducible effect. Figure S2 shows UVB doses-response on HDF and HaCaT cells.

Then, we set ourselves to test whether UV radiations alter cell homeostasis in our system, and whether EDA modulated these effects. To characterize the specific protection conferred by EDA against either UVA or UVB radiation, lamps illuminating with defined spectra were used to pulse-treat cells, which were then further cultured and monitored for 48 h (see Materials & Methods; see demonstrative radiometry in Figure S3). Exposure of each cell type to either UVA or UVB irradiation had a significant impact on cell confluency and morphology (Figure 1A,B, middle columns). This was particularly evident for UVB exposure, whereby a substantial share of cells acquired a rounded, detached appearance (Figure 1). Importantly, pretreatment for 24 h with 0.5 mg/mL EDA robustly reverted these phototoxicity-associated phenotypes in both cell types (Figure 1, rightmost column). Similar observations were recorded when comparing treated cultures for 48h (data not shown). These results supported that EDA acts as an effective photoprotector in fibroblasts and keratinocytes in vitro, and prompted us to get further mechanistic detail.

### 2.2. EDA Attenuates UVR-induced DNA Damage and Associated Stress Signalling in Fibroblasts and Keratinocytes

**Figure 2:**
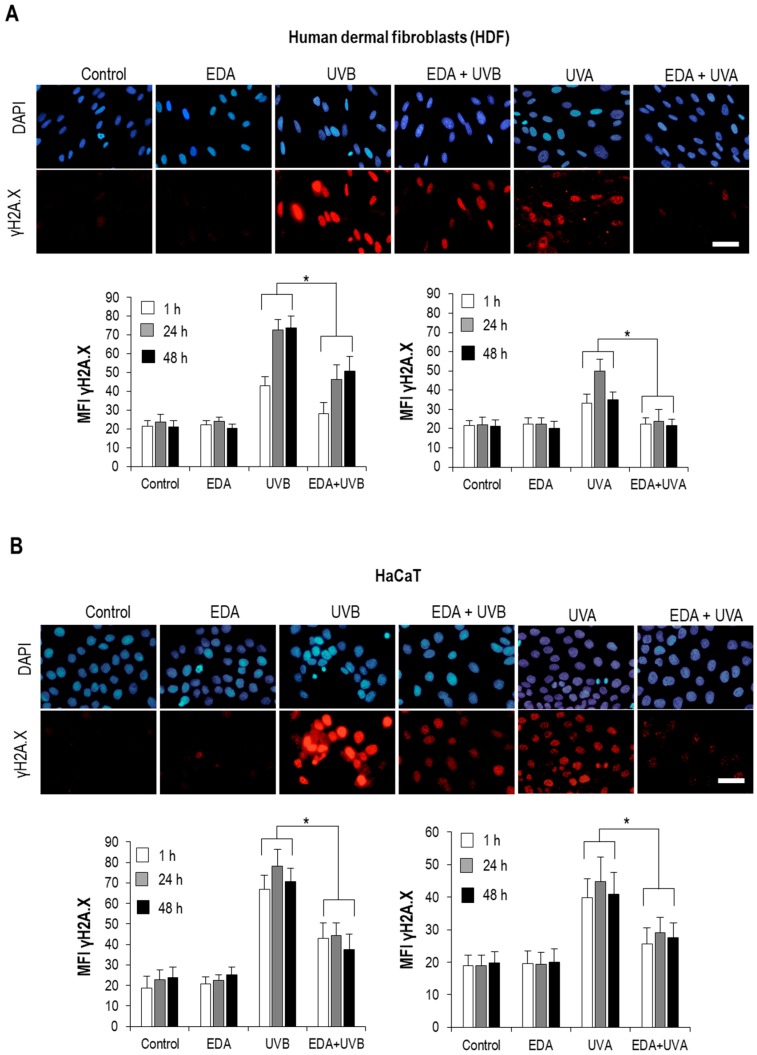
γH2A.X expression on HDF and HaCaT cells after EDA and UV treatment. HDF (**A**) and HaCaT cells (**B**) were incubated with 0.5 mg/mL EDA for 24 h and then irradiated with UVB (700 mJ/cm^2^ for HDF and 300 mJ/cm^2^ for HaCaT cells) or UVA (3000 mJ/cm^2^ for both cell types). γH2A.X expression was determined by immunofluorescence staining after 1, 24 and 48 h UV radiation. Images show γH2A.X expression after 24 h UV radiation in a representative experiment in HDF (**A**) and HaCaT cells (**B**). Graphs show the mean ±SD of MFI of γH2A.X expression in 300 HDF (**A**) and HaCaT cells (**B**) after 1, 24 or 48 h post UVB (left graphs) and UVA (right graphs) irradiation. Scale bar: 20 µm. * *p* ≤ 0.05.

A major feature of photoxicity is the damage of genetic material in the cell. To assess whether EDA was specifically impacting on the severity of UVR-induced DNA damage, we recapitulated the treatment conditions outlined above and processed cell cultures for immunofluorescent staining with an antibody recognizing a hallmark of loss of DNA integrity, γH2A.X, for their analysis by fluorescence microscopy [10]. As expected, exposure of each cell type to either UVA or UVB irradiation led to a significant increase of γH2A.X signal (measured as mean fluorescence intensity, MFI) in nuclei 1, 24 and 48 h post-irradiation, confirming that in our experimental conditions extensive DNA damage is exerted upon irradiation (Figure 2A,B). The γH2A.X signal was also higher upon UVB irradiation, consistent with the higher severity of the phenotypes observed in our morphological analyses (see above). Incubation with EDA alone did not alter the basal γH2A.X signal from the control. However, and in accordance with a substantial photoprotective effect, pretreatment with EDA significantly decreased γH2A.X signal at all tested timepoints (Figure 2A,B).

**Figure 3:**
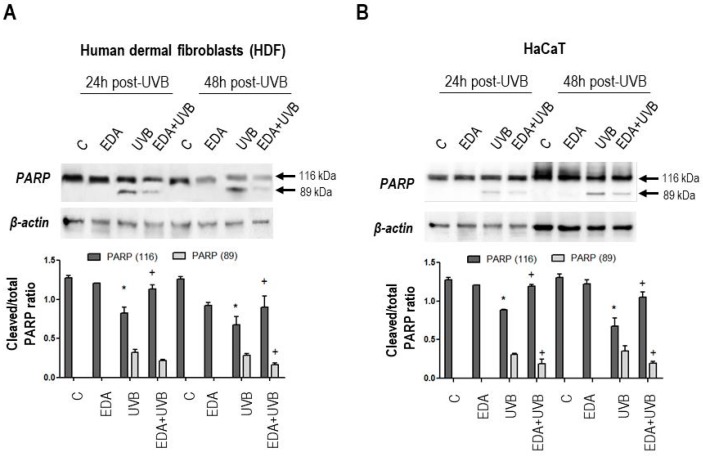
PARP expression in HDF and HaCaT cells after treatment of EDA and UVB. HDF (**A**) and HaCaT cells (**B**) were incubated with EDA (0.5 mg/mL) for 24 h and then irradiated with UVB light (700 and 300 mJ/cm^2^ for HDF and HaCaT cells, respectively). PARP expression was analysed 24 and 48 h post-irradiation by Western blot in HDF (**A**) and HaCaT cells (**B**). Graphs show mean ±SD of cleaved/total PARP ratio in HDF (**A**) and HaCaT cells (**B**) treated under different conditions. β-actin was used as loading control. * *p* ≤ 0.05 regarding to untreated (C) cells; + *p* ≤ 0.05 regarding to UV irradiated cells.

Furthermore, we assessed a signalling node immediately downstream severe DNA damage and γH2A.X accumulation: polyADP-ribose polymerase (PARP) cleavage [11]. As expected for both cell types, the extensive damage caused by UVB irradiation was associated with the cleavage of the active, full-length PARP polypeptide (~116 kDa) yielding a ~89 kDa fragment (Figure 3A,B). Consistent with the previous observations, pretreatment with EDA protected cells from photoxicity and attenuated PARP cleavage (Figure 3A,B), although not completely. In fact, the analysis of cell cycle distribution of HDF cells across the different treatment conditions revealed that checkpoint mechanisms were still active, stalling irradiated cells in the S-G2/M boundary, despite EDA pretreatment (Figure S4). These data confirm EDA as an effective nontoxic compound protecting fibroblasts and keratinocytes from phototoxicity, including DNA damage.

**Figure 4:**
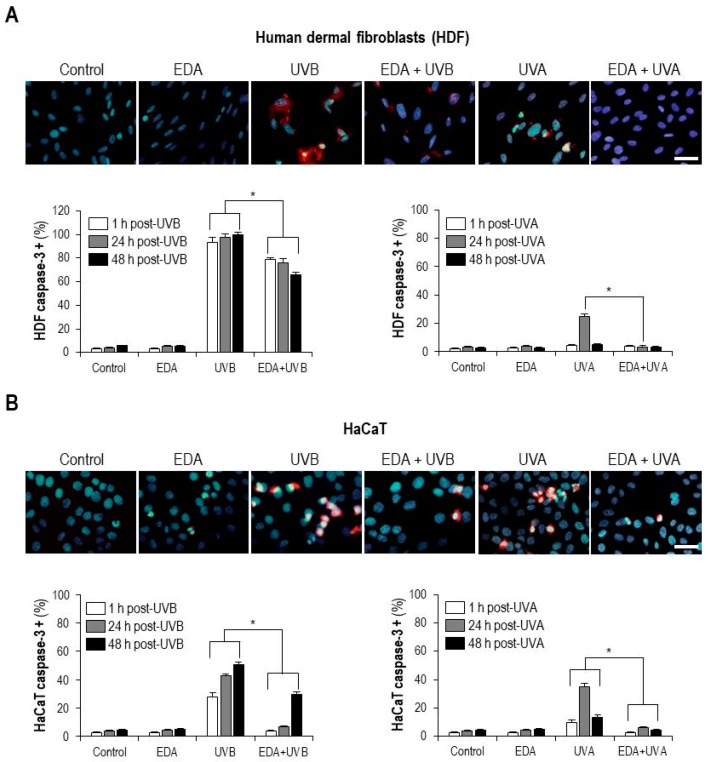
Caspase-3 expression in HDF and HaCaT cells after EDA and UV treatment. HDF (**A**) and HaCaT cells (**B**) were incubated with 0.5 mg/mL EDA for 24 h and then irradiated with UVB (700 mJ/cm^2^ for HF and 300 mJ/cm^2^ for HaCaT cells) or UVA (3000 mJ/cm^2^ for both cell types). Active caspase-3 expression was determined by immunofluorescence staining after 1, 24 and 48 h UV irradiation. Images show active caspase-3 expression after 24 h UV irradiation in HDF (**A**) and HaCaT cells (**B**) from a representative experiment. Graphs show mean ± SD of percentage of active caspase-3 positive HDF (**A**) and HaCaT cells (**B**) in 700 cells analysed. Scale bar: 30 µm. * *p* ≤ 0.05.

To better evaluate the functional implications of these observations, we determined whether the attenuation exerted by EDA on UV-induced DNA damage was reflected on differential patterns of proapoptotic signalling. We first checked, by immunostaining and image analysis, caspase-3 upregulation, a hallmark of extensive loss of integrity of cell [12]. Treatment and processing for immunostaining across treatment conditions revealed that in both HDF and HaCaT cells, either UVA or UVB irradiation led to a significant increase in caspase-3 positive cells (Figure 4A,B). Of note, while EDA supplementation alone did not have any significant impact, EDA pretreatment significantly diminished caspase-3 upregulation in irradiated cells (Figure 4A,B). Analogous results were obtained when the levels of the Inhibitor of Apoptosis (IAP) protein family member survivin [12] were measured in HDF cells and keratinocytes subject to different treatments: EDA pretreatment blunted UV-induced upregulation of survivin (Figure S5). These results support that the protective effects of EDA on cell culture phenotype and DNA damage signalling reflect on prosurvival signalling.

**Figure 5:**
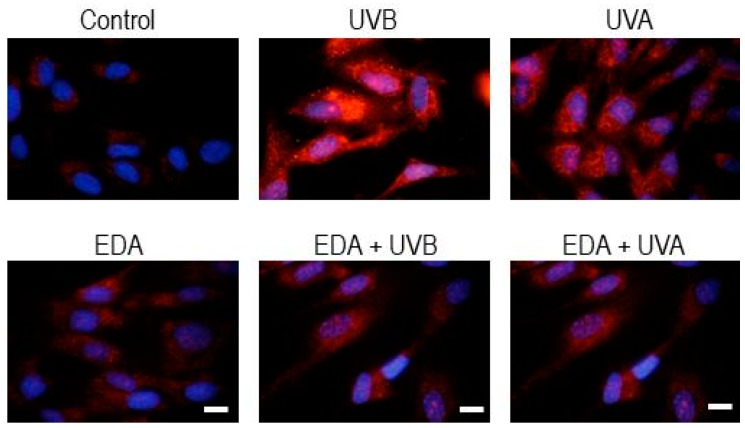
LC3AB expression in HDF after EDA and UV treatment. HDF were incubated with 0.5 mg/mL EDA for 24 h and then irradiated with UVB (700 mJ/cm^2^) or UVA (3000 mJ/cm^2^). LC3AB expression was determined by immunofluorescence staining after 24 h UV irradiation. Scale bar: 10 µm.

As a more generic stress response hallmark, we evaluated the activation state of autophagy in HDF cells across conditions [13]. The upregulation of key components of the autophagic machinery, such as p62 and LC3 is a recurrent target in adaptive stress responses and can be driven by different stress response factors, such as Nrf2 or the PKR-like endoplasmic reticulum kinase (PERK)-dependent branch of the Unfolded Protein response [14,15]. In accordance with a robust photoprotective role of EDA in our experimental setting, while mock-pretreated cells significantly upregulated the pivotal regulator of autophagy Light Chain 3-beta (LC3B) after 24 h upon exposure to UVB and UVA radiation, EDA-pretreated cells did not exhibit such robust upregulation of autophagy signalling (Figure 5). Similar results were observed 48 h post-irradiation (data not shown).

**Figure 6:**
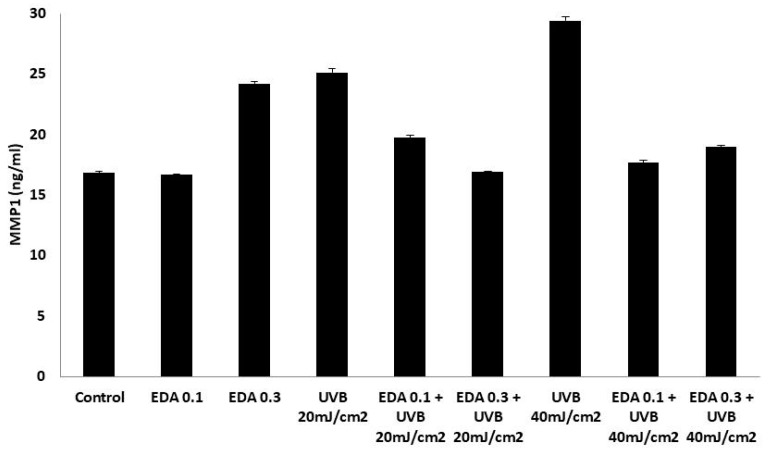
ELISA for secreted MMP1 levels in supernatants from HDF cultures irradiated with UVB. HFD cells, either nonpretreated or exposed for 24 h to two different EDA concentrations (0.1 or 0.3 mg/mL), were left non-irradiated or exposed to UVB radiation at two different dose: 20 and 40 mJ/cm^2^. Cleared supernatants from three biological replicates from each condition were then subject to ELISA assay for MMP1 levels, and the optical absorbance measurements at 405nm interpolated to a standard curve (*r*^2^ = 0.986) to estimate the absolute MMP1 concentration in each sample. Error bars express standard deviation for each triplicate set.

Finally, we evaluated the secretion levels of MMP1 across conditions (Figure 6). Using a colorimetric, enzyme-linked immunoassay, we compared MMP1 levels in culture supernatants of irradiated cells with those of nonirradiated cells in the presence or absence of EDA pretreatment. UVB irradiation elicited a significant increase in secreted MMP1 levels over those of non-irradiated cells, in an energy-dose dependent fashion (Figure 6). Consistent with its protective effect against photoinduced cell damage, pretreatment with EDA abrogated MMP1 upregulation regardless of the irradiation conditions used (Figure 6).

Taken together, our observations support that EDA is a non-toxic compound that confers protection from UV-induced damage of cell structures and downstream signalling, and improves homeostasis and survival of both HDF cells and keratinocytes exposed to ionizing radiation.

### 2.3. Exposure to EDA Protects from TCDD-induced AhR Activation and Toxicity in Fibroblasts and Leratinocytes, and Rescues Loricrin Expression in Keratinocytes

Because toxicity by environmental pollutants is a major aspect of skin damage, aging and tumorigenesis, we decided to explore whether EDA additionally confers protection from the toxic effects of a prototypical environmental pollutant: TCDD, the most common dioxin and a potent tissue damaging agent [16]. First, we tested in vitro a high concentration of TCDD (100 nM) and analysed the survival of HDF cells and keratinocytes at different time points (1.5–48 h) (Figure S6). 100 nM of TCDD had no effect over cell survival at any of the tested time points. Later, in order to determine whether FBS could be affecting the uptake of the dioxin we carried out assays to determine survival of HDF and HaCaT cells after incubation with lower concentrations of TCDD (15, 25 and 50 nM) in the presence of 1–10% FBS. None of the tested conditions had any effect over survival (data not shown) so we next set 10 nM TCDD for 1.5 h for our experiments, as this is the condition more often used in the bibliography to study the translocation of AhR receptor [17,18,19].

**Figure 7:**
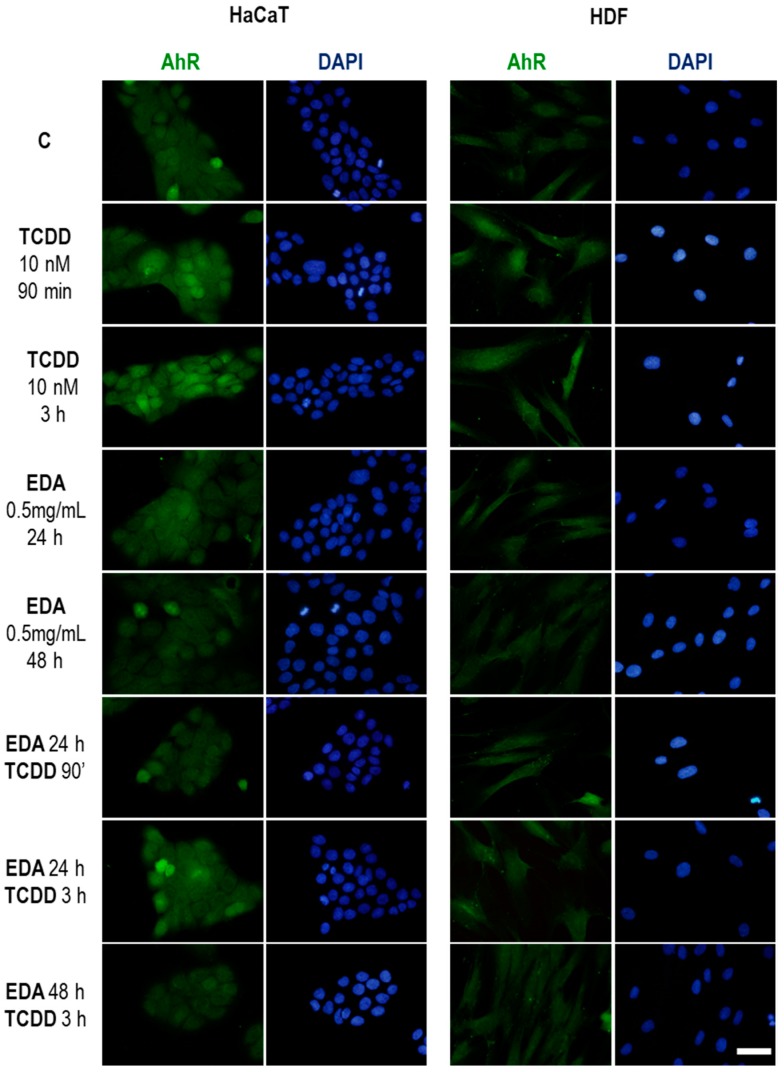
AhR expression on HDF and HaCaT cells after treatment with TCDD and EDA. HaCaT (left panels) and HDF cells (right panels) were treated with EDA (0.5 mg/mL) for 24 or 48 h and then incubated with TCDD (10 nM) for 1.5 or 3 h. AhR expression was analysed by immunofluorescence staining. Images show AhR expression after different combinations of treatments in a representative experiment. Scale bar: 30 μm.

**Figure 8:**
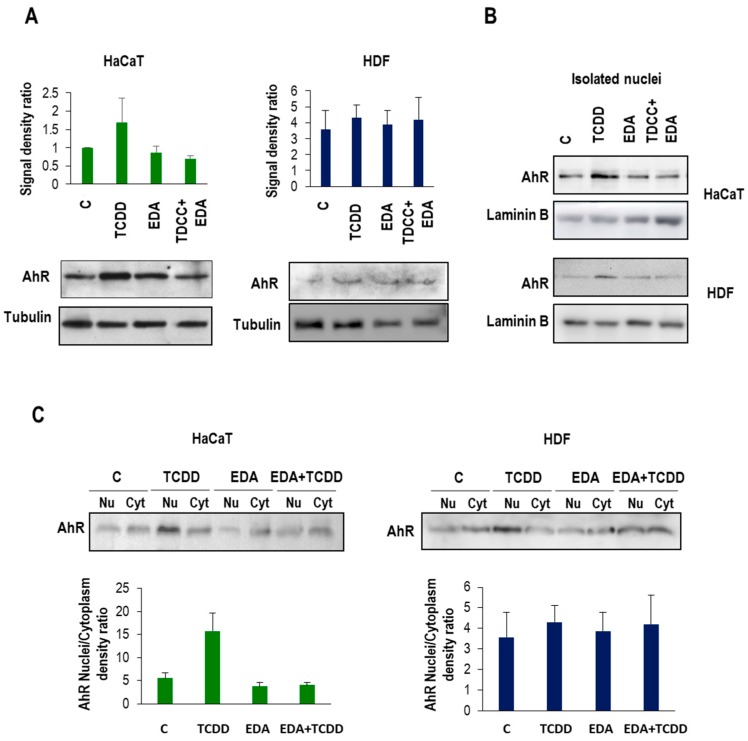
AhR expression in isolated nuclei from HDF and HaCaT cells treated with TCDD and EDA. HDF and HaCaT cells were treated with EDA (0.5 mg/mL) for 24 h and then incubated with TCDD (10 nM) for 1.5 h. (**A**) AhR expression was analysed 24 h post-irradiation on total cell lysates by western blot. Graphs show mean ±SD of signal density AhR/tubulin ratio in two representative experiments. (**B**) AhR expression was analysed 24 h post-irradiation in lysates from isolated nuclei from HaCaT (upper panel) and HDF (lower panel) by western blot. Laminin B was used as loading control. (**C**) AhR expression was analysed 24 h post-irradiation in lysates from nuclei (Nu) and cytoplasm (Cyt) from HaCaT (left panel) and HDF (right panel) by western blot. Graphs show mean±SD of AhR nuclei/cytoplasm ratio in keratinocytes and HDF treated under different conditions. In all cases Image Lab software (Bio-Rad) was used for band quantification.

Exposure to either HDF and HaCaT cells to nanomolar concentrations of TCDD elicited an upregulation of AhR protein (the natural ligand) at times as early as 90min as assessed by epifluorescence microscopy (Figure 7). Importantly, EDA did not display a prominent effect on AhR expression, but abrogated in both cell types the upregulation of the protein exerted by TCDD (Figure 7). Furthermore, western blot analysis of isolated nuclear and cytoplasmic fractions demonstrated that EDA pretreatment blunted the nucleocytoplasmic shuttling of AhR, which is increased by exposure to TCDD (Figure 8). These experiments demonstrate that EDA is capable of counteracting TCDD-associated AhR upregulation and activation.

**Figure 9:**
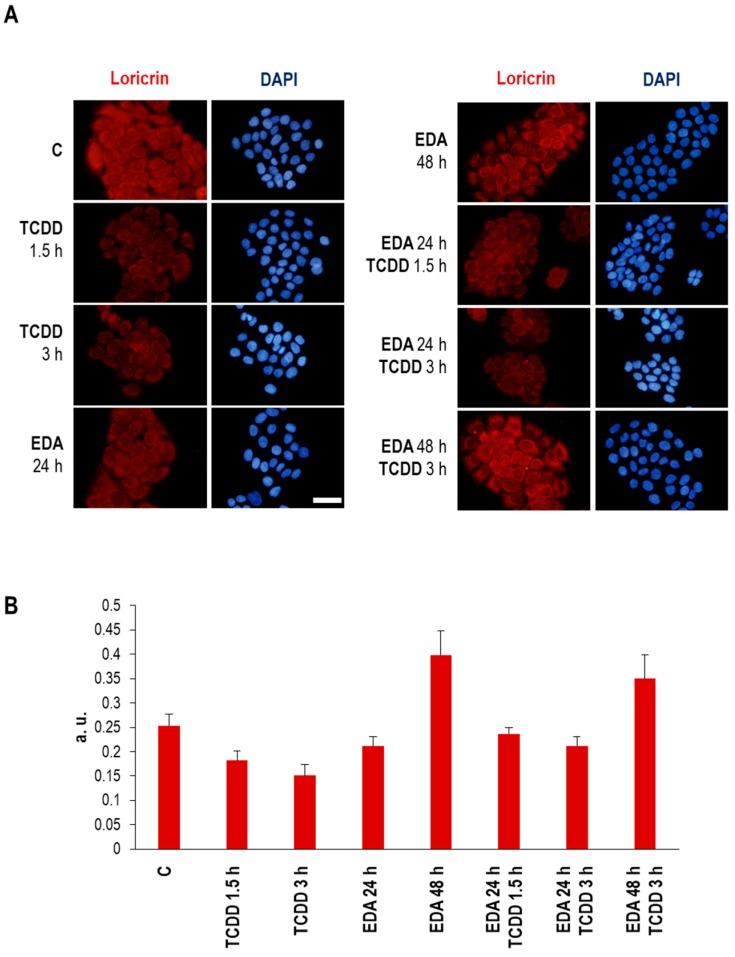
Loricrin expression in HaCaT cells treated with TCDD and EDA. HaCaT cells were treated with EDA (0.5 mg/mL) for 24 or 48 h and then incubated with TCDD (10 nM) for 1.5 or 3 h, and loricrin expression was determined by immufluorescence staining. (**A**) Images show loricrin expression in HaCaT cells from a representative experiment. Scale bar: 30 μm. (**B**) Graph shows mean ±SD of MFI of loricrin in 300 analysed cells for each condition.

Finally, we assessed the functional impact of abrogating TCDD exposure-associated signalling upon EDA supplementation. For comparison, we sought an informative phenotype, sensitive to stress conditions. Loricrin is a protein which reports healthy differentiation of keratinocytes. Ionizing radiations can affect its expression, reflecting alterations of cell homeostasis [20]. Importantly, exposure of HaCaT cells to TCDD promoted a loss of loricrin staining which was reverted upon previous exposure to EDA (Figure 9). Of note, extended exposure to EDA for 48 h was sufficient per se to induce an upregulation of loricrin expression. This fact suggests that treatment with EDA compound not only protects from TCDD toxicity, but promotes a state further apart from the damaged phenotype.

Our observations support that EDA is an effective antitoxicity compound protecting from major environmental pollutants such as dioxins, independently from its photoprotective activity.

## 3. Discussion

Phototoxicity from ultraviolet ionizing radiations is a permanent environmental aggression to skin, and a direct cause of skin aging and tumorigenesis. Different factors derived from modern lifestyles (increased age expectancy; promotion of outdoor activity and travelling to different latitudes; increasing presence of environmental contaminants with toxic potential) challenge skin health and require the development of technologies that minimize their impact. Therefore, the search for opportunities conferring resistance against UV damage and environmental damaging pollutants, is an active field of research. Natural extracts and compounds are particularly interesting, because they have typically evolved low toxicity and high anti-stressor activities, and very often combine multiple synergistic effects such as antioxidant and enhancer of tissue repair. In this study, we report the capacity of a hydrophilic extract from *Deschampsia antarctica* (EDA) to reduce toxicity induced by UV light on skin fibroblasts and keratinocytes as inferred from different molecular markers, including DNA damage-associated γH2AX, PARP cleavage, induction of autophagy and secretion of MMP1. Of note, EDA was also capable of blunting the toxicity of TCDD, a potent dioxin that is known to amplify the damage and subsequent carcinogenic effect of other agents [16].

The inhibition of TCDD toxicity by EDA was not only evaluated through indirect proxies for skin cell homeostasis (proliferation, morphology, loricrin expression) but also directly by assessing the expression levels and translocation of the TCDD receptor in the cell, AhR. While EDA could boost degradation mechanisms targeting TCDD independently of AhR, it is tempting to speculate that EDA exerts direct regulation on AhR, possibly through proteostatic mechanisms such as Hsp90, given its anti-inflammatory activity in vitro [21]. Of note, these mechanisms might also contribute to the protective effect of EDA against phototoxicity.

Generic mechanisms such as antioxidant activity from phenolic compounds in EDA could partly explain a blockade of DNA damage and downstream responses [22], because dysregulated ROS accumulation is both a hallmark of phototoxicity as well as a major cause for the oxidative damage of genomic material and other structures such as lipids. Nonetheless, a non-exclusive protective mechanism likely at play is the direct quenching of ionizing radiation, because apart from adaptations of its photosynthetic machinery, *Deschampsia antarctica* plants produce substantial amounts of carotenoids such as zeaxanthin and other xanthophyll cycle intermediates [23], which are known to confer photoprotection. Future studies may characterize isolate contributions from different components in EDA to specific mechanisms of cell protection.

Interestingly, our observations support that while UV-induced apparent DNA damage is significantly diminished by simultaneous exposure to EDA, checkpoint mechanisms are still active and cells undergo blockade of their progression through the cell cycle when exposed to UVB light regardless of the presence or absence of EDA. Specifically, analysis of unsynchronized cell cultures revealed that UVB light elicited an accumulation of cells in S and G2/M phases, and simultaneous exposure to EDA did not significantly diminish this effect. Although the precise mechanisms at play will require extensive mechanistic characterization, beyond the scope of this report, it is clear that certain mechanisms sensing and transducing the impact of UVB on the cell are active despite the presence of EDA. While our assays monitoring stress signalling such as PARP cleavage and autophagy clearly show a protective effect from EDA, a possibility exists that surges in ROS are only partially lowered to levels significantly less harmful, but nonetheless detectable by specific mechanisms such as sestrins [24]. Another non-exclusive explanation would lie on the hypothesis that certain molecules in the cell distinct from DNA are still damaged despite the presence of EDA; a good candidate would be membrane lipids, which face UV radiation at peripheral regions of the cell [25]. Beyond the specific mechanisms at play, our observations in this regard bear relevance because they highlight that checkpoint mechanisms are still effective in cells undergoing stress protection by EDA, a feature of particular importance when considering the antitumoral effect from these preparations.

In summary, we contribute evidence that EDA displays significant protective roles in both fibroblasts and keratinocytes in the face of both UV radiation and prototypical dioxin toxicity, while having little intrinsic impact on cell proliferation and homeostasis. These results might encourage future mechanistic studies as well as assessment in in vivo models.

## 4. Materials and Methods

### 4.1. Reagents

*Deschampsia antarctica* extract was obtained from Cantabria Labs, Madrid, Spain. The methods for obtaining the extract have been previously published [7]. Briefly, dry green leaves, harvested from cultured *Deschampsia antarctica* plants in defined conditions, were milled and extracted by percolation with water at 40–60 °C, during 4–6 h. The extract was filtered through a 1μm filter and lyophilized. This stock was prepared in the culture medium to the desired concentration.

Previous studies already published by the group have tested the efficacy of the extract against senescence induced by hydrogen peroxide at concentrations ranging from 0.3 through to 1 mg/mL [7]. Based on these studies, we treated fibroblasts and keratinocytes under the following conditions: 0.5 mg/mL of EDA for 24 h before exposition to environmental aggressive agents. For detection of MMP1, HDF were treated with 0.1 and 0.3 mg/mL EDA for 24 h.

### 4.2. Cell Cultures

Human dermal fibroblasts (HDF) were obtained from a skin biopsy after two rounds of trypsinization with 0.25% Trypsin-EDTA by shaking at 37° C. We also used the keratinocyte cell line HaCaT. Cells were cultured in Dulbecco’s modified eagle medium (DMEM) supplemented with 10% (*v*/*v*) fetal bovine serum (FBS) and penicillin (50 μg/mL) and streptomycin (50 μg/mL) (HyClone Laboratories, South Logan, UT, USA). Cells were maintained at 37 °C, 95% humidity and 5% CO_2_ in an incubator (Heraeus HERAcell, Thermo Scientific, Waltham, MA, USA).

### 4.3. Irradiation

HDF and HaCaT were subjected to UVA and UVB radiation. The sources of UVA and UVB lights were a lamp of 300–400 nm and 15.9 W/m^2^ (CAMAG) and a lamp of 270–380 nm, which was filtrated by glass filter of 305 ± 5 nm (Newport, Irvine, CA, USA) respectively. The spectra of the UVA and UVB lamps were recorded and measured using a radiometer USB2000+ (ocean Optics, Dumedin, FL, USA) (Figure S3). UV dose-response curves were carried out (Figure S2) and different doses were used in most of the assays: 700 mJ/cm^2^ and 300 mJ/cm^2^ in the case of UVB for HDF and HaCaT cells respectively, and 3000 mJ/cm^2^ in the case of UVA for both cell types. For MMP1 detection assays HDF cells were irradiated with UVB light at lower dose: 20 and 40 mJ/cm^2^, to induce detectable cell stress while avoiding the triggering of proapoptotic signalling.

For irradiation, cell culture medium was replaced by PBS in all cases. Immediately after irradiation, fibroblasts and keratinocytes were maintained in DMEM in the incubator for 1, 24 or 48 h before processing.

### 4.4. Dioxin: Tetrachlorodibenzo-p-dioxin (TCDD)

Different concentrations of TCDD (15–300 nM) were tested on human dermal fibroblasts and keratinocytes at different incubation times, always without exceeding a final DMSO concentration of 0.1% (Sigma, Saint Louis, MO, USA).

### 4.5. Cell Morphology and Viability Analysis

Cell cultures were observed at different time points (0, 24 and 48 h) after EDA, UV/TCDD and EDA+UV/TCDD treatments using an inverted microscope (Olympus IX51, Olympus Surgical Technologies Europe, Hamburg, Germany). Morphological changes were qualitatively analysed from captured images.

Cell viability in HaCaT and HDF cells after different treatments was determined by MTT assay. For that, cells 24 h after the treatments, were incubated with MTT (50 μg/mL) for 3 h. The formazan crystals were dissolved with DMSO and the optical density was determined in a SpectraFluor plate reader (Tecan Trading AG, Switzerland) at a wavelength of 542 nm.

Cell proliferation in HaCaT and HDF cells after different treatments was measured by crystal violet staining (0.2% (*w*/*v*) in 2% Ethanol) for 20 min. Optical density was determined in a SpectraFluor plate reader (Tecan) at a wavelength of 570 nm.

### 4.6. Immunofluorescence Assays

Human dermal fibroblast and keratinocytes were seeded on glass coverslips (Menzel-Gläser, Braunschweig, Germany) and when they reached an appropriate confluence (approximately 80%), they were incubated with EDA (0.5 mg/mL) for 24 h. Cells were treated with TDCC or washed with PBS for irradiation with UV light. After treatment, cells were fixed with 3.7% formaldehyde (Panreac, Barcelona, Spain), and then permeabilized with 0. 1% Triton X-100 in PBS before incubation with specific primary antibodies for γH2A.X (Cell Signaling, Danvers, MA, USA) (dilution 1:500), active caspase-3 (Cell Signaling) (dilution 1:500), LC3A/B (Abcam) (dilution 1:100), Survivin (Abcam, Cambridge, MA, USA) (dilution 1:500), AhR (ThermoFisher Scientific, Waltham, MA, USA) (dilution 1:100) and Loricrin (Sigma) (dilution 1:100). Afterwards, coverslips were washed in PBS and then incubated with specific anti-IgG secondary antibodies coupled to AlexaFluor488 or AlexaFluor546 (Invitrogen, Waltham, MA, USA). Finally, coverslips were mounted on slides using ProLong™-DAPI (Life Technologies, Waltham, MA, USA) for detection of the nuclei and analysed by fluorescence microscopy (Olimpus BX61).

### 4.7. Western Blot Assays

For the detection of nuclear AhR and laminin B (Dallas, TX, USA), the extract of the cytoplasmic and nuclear proteins was performed separately with the Nuclear Extraction Kit (Merk) according to manufacturer’s instructions. For the detection of total AhR, Tubulin, PARP and β-actin, extracts were obtained by using the Bio-world Protein Extraction Buffer with phosphatase inhibitors (Roche, Basel, Switzerland) and proteases (Roche), following the manufacturer’s instructions.

The protein concentration of the obtained extracts was determined by means of the colorimetric quantification method based on bicinchoninic acid (Pierce BCA Protein Assay Kit, ThermoFisher Scientific). Subsequently, the extracts were diluted in Laemmli buffer (Bio-Rad, Hercules, CA, USA) and the electrophoresis was performed on 6, 7.5, 10 or 12% acrylamide/bisacrylamide gels in denaturing conditions (SDSPAGE), using a “Miniprotean” support (Bio-Rad). 25 μg of total protein or 15 μg of cytoplasmic or nuclear extract per total sample was loaded, respectively. Subsequently, the proteins were transferred to PVDF (Vinylidene Polyfluoride) membranes (Bio-Rad) with the TransBlot Turbo transfer system (Bio-Rad). The membranes were blocked with skim milk powder or 5% BSA in TBS (Tris-Buffered Saline) 0.1% Tween^®^ 20, according to the requirement of the antibody, for at least 1 h at room temperature under agitation. They were then incubated with the corresponding primary antibody anti-AhR (Thermo Fisher Scientific, Waltham, MA, USA) (dilution 1:250), anti-laminin B (Santa Cruz) (0.5 μg/mL), anti-tubulin (Sigma) (dilution 1:1000), anti-PARP (Cell Signaling) (dilution 1:500), and anti-β-actin (Santa Cruz Ltd.) (2 μg/mL) diluted in the same blocking solution overnight at 4° C under agitation, washed with 0.1% TBS-Tween^®^ 20 and incubated with secondary mouse or rabbit antibody coupled to peroxidase (Thermo Fisher Scientific) for 2 h at room temperature. The development was carried out by chemiluminescence (ECL Plus kit, GE HealthcareChicago, IL, USA) using the ChemiDocTR XRS + high definition system (Bio-Rad). The bands corresponding to the different proteins were digitized using the Image Lab software version 3.0.1 (Bio-Rad).

### 4.8. Analysis of Cell Cycle

Near confluent HDF monolayers were trypsinized 24 and 48 h after treatment (EDA and/or UVB/A) and processed using a DNAprep kit (Qiagen, Hilden, Germany) according to manufacturer’s recommendations. Cellular cycle was evaluated by flow cytometry.

### 4.9. ELISA

MMP1 was analysed in supernatants from cultures of HDF treated with 0.1 or 0.3 mg/mL EDA for 24 h and then irradiated with UVB light at different dose: 20 and 40 mJ/cm^2^. Supernatants were collected 24 and 48 h post-treatment and MMP1 was analysed by ELISA assay (Becton & Dickinson, Franklin Lakes, NJ, USA).

### 4.10. Image Analysis

Microscopic images were obtained using an epifluorescence microscope coupled to a CCD camera DP70 (Olympus BX-61, Tokyo, Japan) with UV filters for the excitation light (360–370 nm excitation filter UG-1), blue (450–490 nm excitation filter BP 490), or green (570–590 nm excitation filter 590 DM). Picture processing was performed with Photoshop Extended CS5 12.0 (Adobe Systems Inc., Mountain View, CA, USA). Both percentages of positive cells and mean fluorescent intensity for specific antibodies were determined by using the Image J analysis software.

### 4.11. Statistical Analysis

Data are represented as the mean standard deviation (SD) of at least three independent experiments. Statistical significance was determined by statistical test of analysis of variance (ANOVA) or two-way ANOVA and Bonferroni post hoc tests, using GraphPad Prism 5.00 (GraphPad Software, Inc., San Diego, CA, USA). Differences were considered to be significant when *p* < 0.05.

## Supplementary Materials

Supplementary Materials can be found at https://www.mdpi.com/1422-0067/20/6/1356/s1.

## Abbreviations

AhR	Aryl hydrocarbon receptor
ANOVA	Analysis of variance
a. u.	Arbitrary units
ECM	Extracellular matrix
EDA	*Deschampsia antárctica*
FBS	Fetal bovine serum
TCDD	2,3,7,8-Tetrachlorodibenzo-p-dioxin
TIMPs	Inhibitors the tissue inhibitor of metalloproteinases
DMEM	Dulbecco’s modified eagle medium
HaCaT	Human keratinocyte cell line
HDF	Human dermal fibroblasts
IAP	Inhibitor of Apoptosis
LC3B	Light Chain 3-beta
MFI	Mean fluorescence intensity
MMPs	Matrix metalloproteinases
PARP	PolyADP-ribose polymerase
PERK	PKR-like endoplasmic reticulum kinase
ROS	Reactive oxygen species
SD	Standard deviation
UV	Ultraviolet light
UVA	Ultraviolet light A
UVB	Ultraviolet light B
UVR	Ultraviolet ionizing radiation
