# Supplementary material for: Extract of Deschampsia antarctica (EDA) Prevents Dermal Cell Damage Induced by UV Radiation and 2,3,7,8-Tetrachlorodibenzo-p-dioxin
Source: Int J Mol Sci. 2019 Mar 18;20(6):1356. doi: 10.3390/ijms20061356 (PMC6471785; doi:10.3390/ijms20061356)
Supplement: Supplementary file 1 [file ijms-20-01356-s001.zip › Supplementary legends.docx]

**Figure S1. EDA *per se* effect over proliferation of HDF and HaCaTcells.** Cells were incubated with different concentrations of EDA (0,05-1 mg/ml) for 24 and 48 hours. After the incubation time viability was measured by MTT assay *(⁎ p<0.05).* Graphs show mean±SD of the percentage of proliferation of treated HDF **(A)** and HaCaT cells **(B)** relative to untreated (0) cells’ proliferation.

**Figure S2. Spectra of the UVA and UVB lamps.** Power density (W/m2) and wavelength (nm) of the UVA (**A**) and UVB (**B**) lamps were recorded and measured using a radiometer USB2000+ (Ocean Optics, Dumedin Florida, USA).

**Figure S3. Cellular cycle on post-treated HDF.** HDF were incubated with EDA (0,5 mg/ml) for 24 h and then irradiated with UVB (700 mJ/cm^2^) **(A-D)** or UVA (3000 mJ/cm^2^) **(E-H)**. Cellular cycle was analysed by flow cytometry 24 and 48 h post-treatment. **p*≤0.05 regarding to untreated cells (Control); +*p*≤0.05 regarding to UV irradiated cells.

**Figure S4. Survinin expression on UVA and EDA treated HDF and HaCaT cells.** HDF **(A)** and HaCaT cells **(B)** were incubated with EDA (0,5 mg/ml) for 24 h and then irradiated with UVA (3000 mJ/cm^2^). The expression of survinin was analysed 24 and 48 h post-irradiation by immunofluorescence. 600-800 cells were analysed for each condition. Scale barr: 30 µm. Images show a representative experiment on HDF **(A)** and HaCaT cells **(B)** 24 h post-irradiation. Left graphs show mean±SD of HDF **(A)** or HaCaT cells **(B)** positive for survinin for each condition 24 or 48 h post-irradiation. Right graphs show mean±SD of MFI of survinin on HDF **(A)** or HaCaT cells **(B)** for each condition 24 or 48 h post-irradiation. ***p*≤0.005; ****p*≤0.001

**Figure S5: MTT assay for treatment of TCDD.** HDF and HaCaT cells were treated with 100 nM TCDD for different periods of time (1.5-48 hours). After the incubation time viability was measured by MTT assay. **(A)** Images were taken at different time points. **(B)** Graphs show mean±SD of the percentage of proliferation of treated HaCaT and HDFs relative to untreated (Ct, 100% survival).

**Figure S6: MTT and Crystal Violet assays in HDF and HaCaT cells irradiated with UVB.** MTT (left graphs, % survival) and Crystal Violet (right graphs, % proliferation) assays in HaCaT **(A)** and HDF **(B)** cells irradiated with different dose of UVB light. Cell proliferation and survival were estimated in percentage with respect to absorbance measurements 24 hours post-ir radiation. Images show HaCaT **(A)** and HDF **(B)** cells 24 hours post-irradiation with different doses of UVB light. Graphs show mean±SD of the percentage of survival and proliferation of irradiated HaCaT and HDF cells relative to untreated (Non-irradiated, 100% survival or proliferation) from triplicates od a representative experiment.
