# Supplementary figures and images for: Extract of Deschampsia antarctica (EDA) Prevents Dermal Cell Damage Induced by UV Radiation and 2,3,7,8-Tetrachlorodibenzo-p-dioxin
Source: Int J Mol Sci. 2019 Mar 18;20(6):1356. doi: 10.3390/ijms20061356 (PMC6471785; doi:10.3390/ijms20061356)

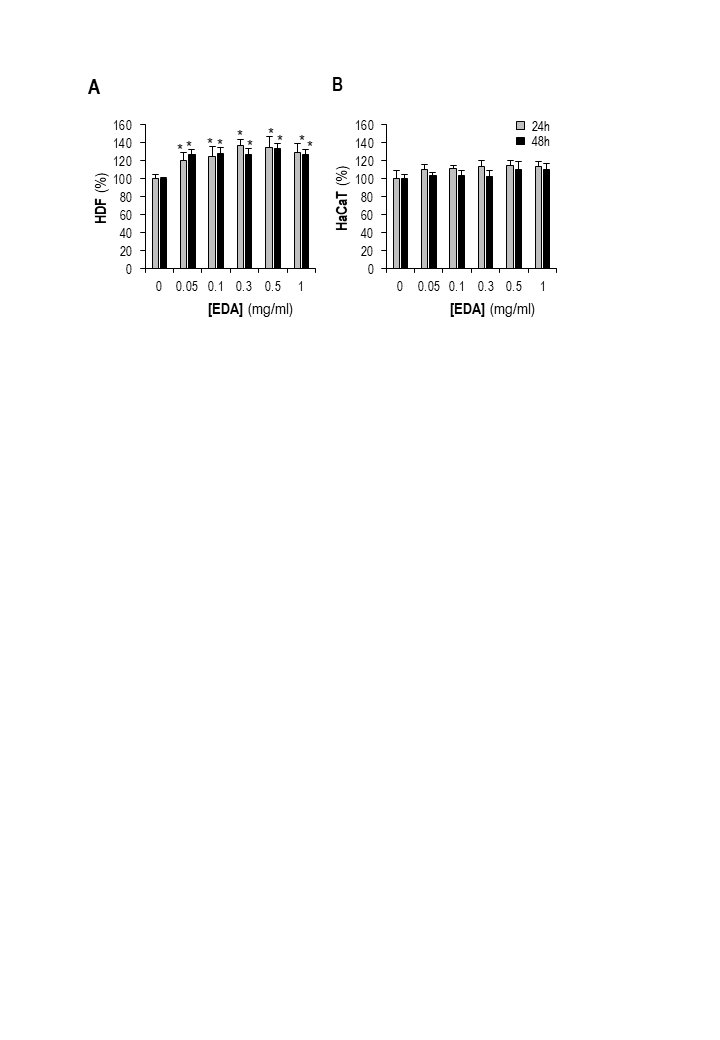

Supplement: Supplementary file 1 [file ijms-20-01356-s001.zip › Fig S1. EDA per se effect over proliferation of HDF and HaCaT.tif]

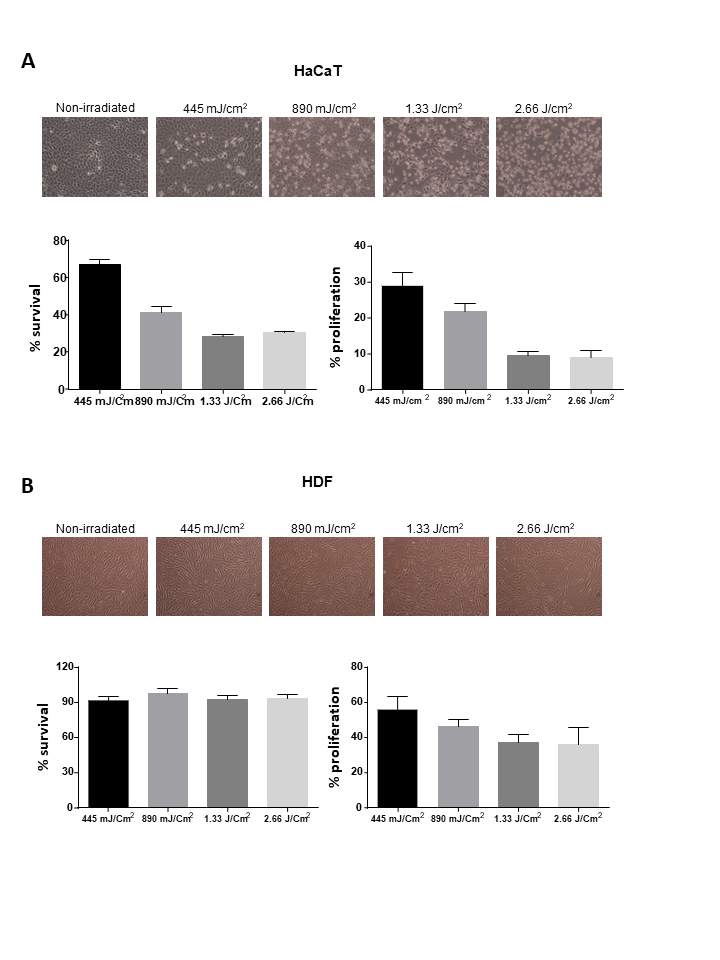

Supplement: Supplementary file 1 [file ijms-20-01356-s001.zip › Fig S2. Dose-response curves for UVB irradiation in HDF and HaCaT cells.tif]

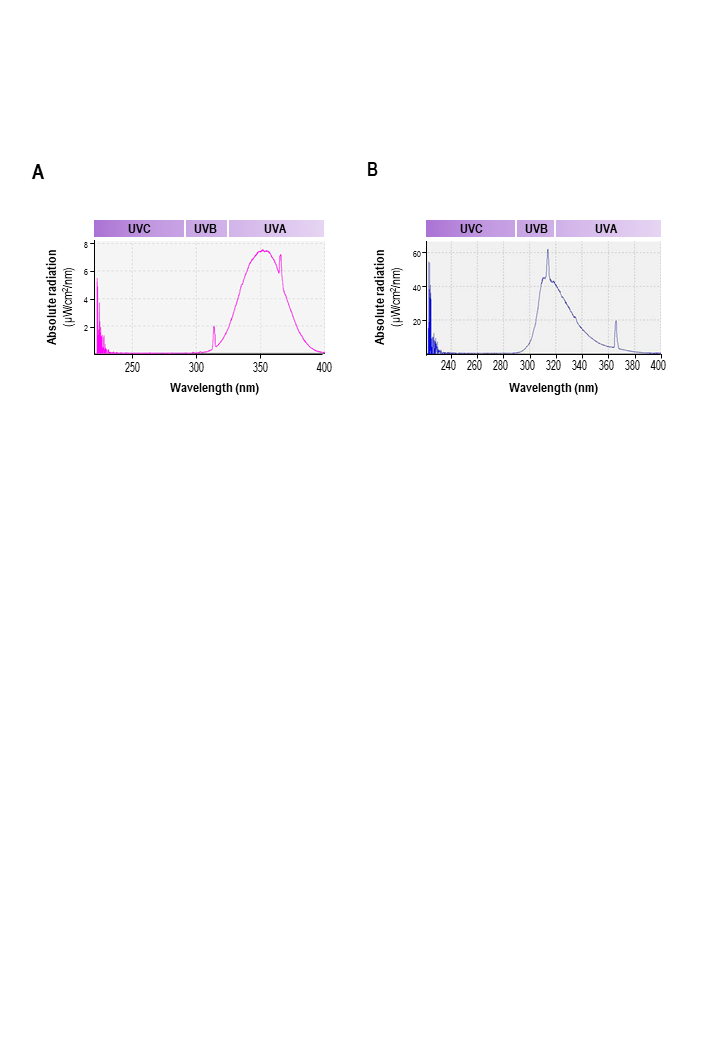

Supplement: Supplementary file 1 [file ijms-20-01356-s001.zip › Fig S3. Spectra of the UVA and UVB lamps.tif]

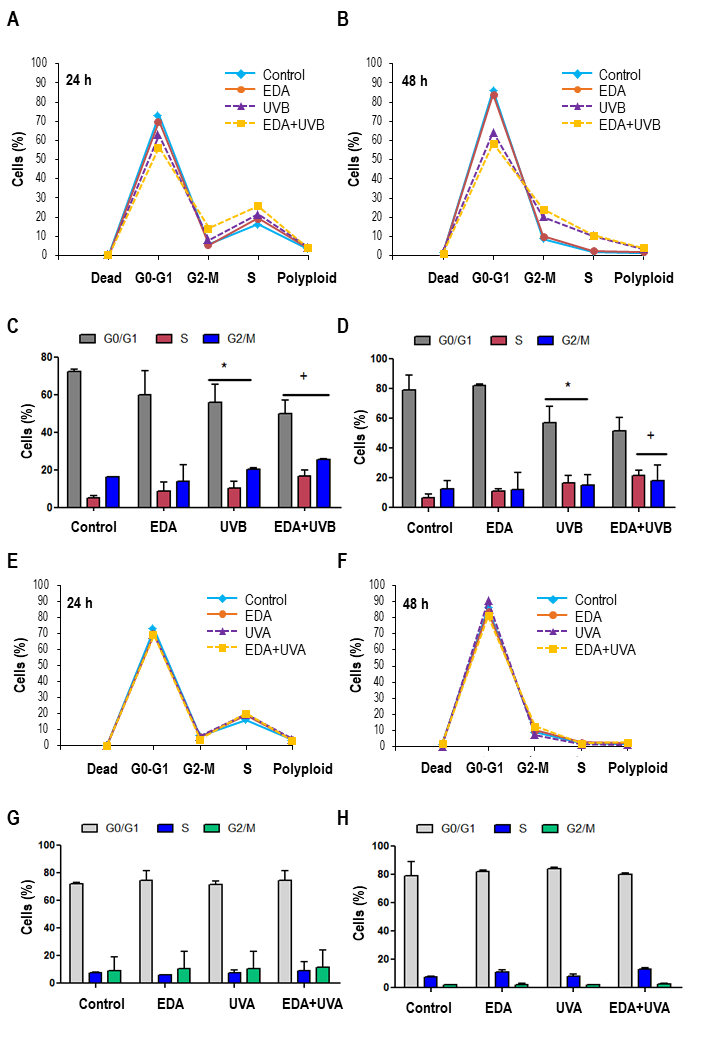

Supplement: Supplementary file 1 [file ijms-20-01356-s001.zip › Fig S4. Cellular cycle on post-treated HDF.tif]

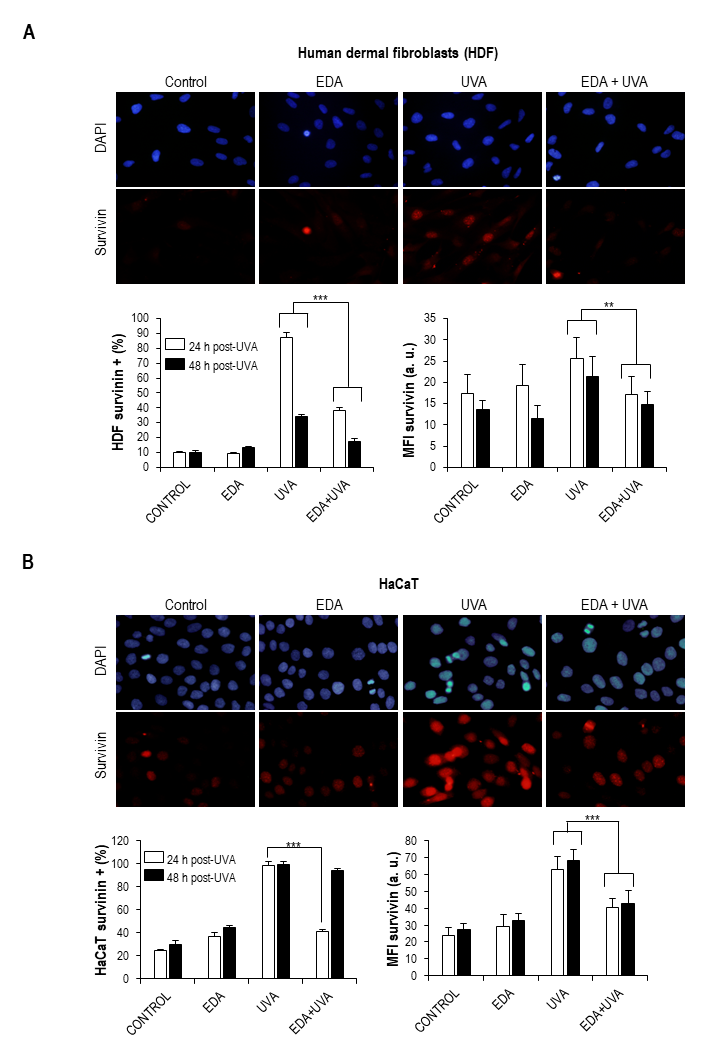

Supplement: Supplementary file 1 [file ijms-20-01356-s001.zip › Fig S5. Survinin expression on UVA+EDA treated HDF and HaCaT cells.tif]

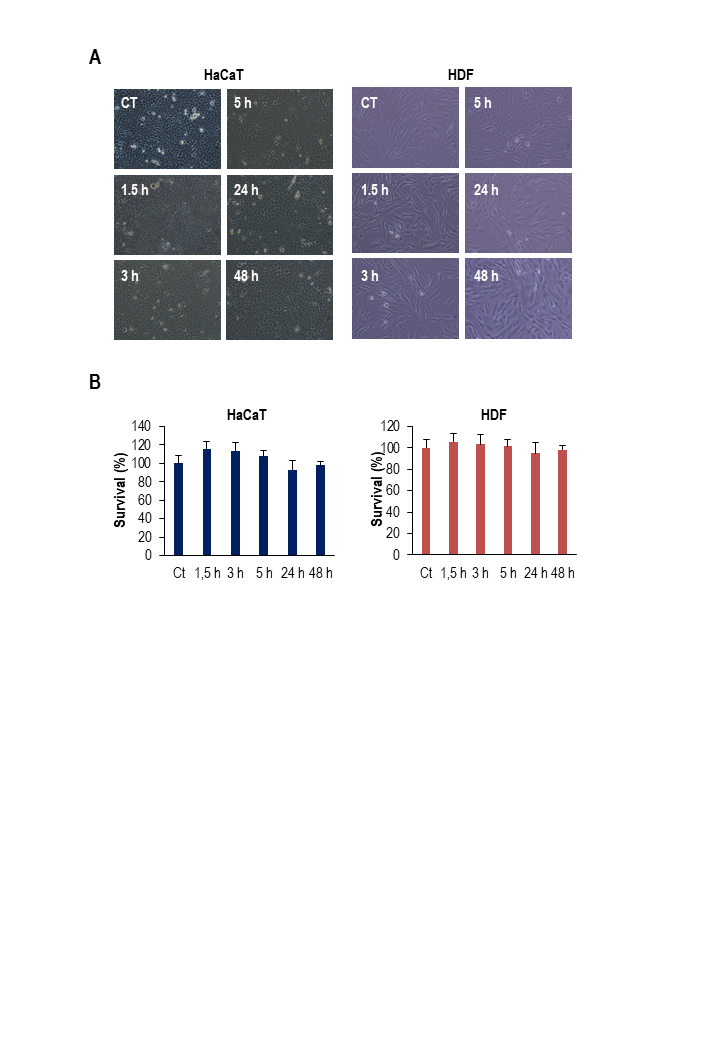

Supplement: Supplementary file 1 [file ijms-20-01356-s001.zip › Fig S6. MTT assay for treatment of TCDD.tif]
